# College and Hospital Notes

**Published:** 1899-04

**Authors:** 


### COLLEGE AND HOSPITAL NOTES.

Dr. Marie J. Mergler has been elected dean of Northwestern University Woman's Medical School, in place of Dr. I. N. Danforth, resigned. Dr. Danforth has been elected dean emeritus. The yearly course at this school has been changed from one of two semesters to one of four semesters of twelve weeks each, commencing the 1st of July, October, January and April. Three semesters will be required; the other semester will be optional. The number of regular students will be limited to 100, twenty-five in each class. They will be admitted to competitive examination for place in class, only after having complied with the requirements of the State Board of Health.

---

The annual commencement of the Medical Department of the University of Buffalo will be held Tuesday, April 25, 1899. The executive committee of the Alumni Association is making an effort to assemble the members by classes and class officers have been appointed to facilitate the work. Dr. E. N. Brush, president of the class of '74 desires all members of this class to correspond with him on the subject, and beyond all to diligent effort to attend. Dr. Brush is superintendent of the Sheppard and Enoch Pratt Hospital, and his address is Towson P. O., Baltimore county, Md.

By the will of the late Elizabeth H. Gates, the Buffalo General Hospital receives the munificent bequest of $50,000 with the additional sum of $1,000 for the continued maintenance of the room in the hospital of which she had charge. Among the several charitable bequests to other institutions are the following: To the Church Charity Foundation, $5,000; to the Home of the Friendless, $5,000; to the Children's Hospital, $6,000, and to the University of Buffalo, for the use of the medical department, $5,000.

Besides these bequests for medical charities she also generously makes another bequest to the Buffalo Fine Arts Academy of $50,000, to be known as the Elizabeth H. Gates Fund, and the interest of which is to be applied to the purchase of works of art by foreign artists. She also gives to the Buffalo Fine Arts Academy all her excellent collection of oil paintings, other than family portraits, and these she desired, if possible, to have hung in connection with those which shall be bought from the interest of the fund she bequeaths to the Academy and she desired to have the whole collection known as the Elizabeth H. Gates collection.

Thus the good work of a noble woman is continued after her death for the relief and succor of the needy and afflicted and for the enjoyment and edification of those more fortunately situated.

---

The New York Post-Graduate Hospital is to have a new training school for nurses, the gift of Harris C. Fahnestock, and it will cost $100,000, supplanting the old one, which has become inadequate to the needs of the hospital.

The new school will be known as the Margaret Fahnestock Training School for Nurses of the New York Post Graduate Hospital. Mr. Fahnestock is founding the school in memory of his wife, and it is to bear her name. It seems that the last days of Mrs. Fahnestock's life were cheered and brightened by the tender care and ministrations of two trained nurses. Mr. Fahnestock took a deep interest in their work and realised how much they had done to make the last days of his wife less of a burden and the gift is a tribute to them and their calling, and in memory of Mrs. Fahnestock.

---

The German Hospital being erected on Jefferson street, near Genesee, is fast approaching completion and will be an ornament to that portion of the city as well as a noble institution for the alleviation and restoration of the afflicted poor.

THE board of managers of the German Deaconess Hospital, on Kingsley street, intend building an addition to the hospital, to meet the increasing demands made upon this highly successful institution.

THE Riverside Hospital, 306 and 308 Lafayette avenue, is soon to be materially enlarged and supplied with all the modern hospital improvements, making it one of the most complete and comfortable of private hospitals to be found anywhere. It is expected that the new addition will be ready by mid-summer.

